# Mechanisms of phosphatidylserine influence on viral production: A computational model of Ebola virus matrix protein assembly

**DOI:** 10.1016/j.jbc.2022.102025

**Published:** 2022-05-11

**Authors:** Xiao Liu, Ethan J. Pappas, Monica L. Husby, Balindile B. Motsa, Robert V. Stahelin, Elsje Pienaar

**Affiliations:** 1 Weldon School of Biomedical Engineering, Purdue University, West Lafayette, Indiana, USA; 2 Department of Medicinal Chemistry and Molecular Pharmacology, Purdue University, West Lafayette, Indiana, USA

**Keywords:** computational biology, mathematical modeling, Ebola virus, virus assembly, phosphatidylserine, VP40, BSL, biosafety level, EBOV, Ebola virus, EVD, EBOV disease, ODE, ordinary differential equation, PM, plasma membrane, PRCC, partial rank correlation coefficient, PS, phosphatidylserine, SPR, surface plasmon resonance, VLP, virus-like particle

## Abstract

Ebola virus (EBOV) infections continue to pose a global public health threat, with high mortality rates and sporadic outbreaks in Central and Western Africa. A quantitative understanding of the key processes driving EBOV assembly and budding could provide valuable insights to inform drug development. Here, we use a computational model to evaluate EBOV matrix assembly. Our model focuses on the assembly kinetics of VP40, the matrix protein in EBOV, and its interaction with phosphatidylserine (PS) in the host cell membrane. It has been shown that mammalian cells transfected with VP40-expressing plasmids are capable of producing virus-like particles (VLPs) that closely resemble EBOV virions. Previous studies have also shown that PS levels in the host cell membrane affects VP40 association with the plasma membrane inner leaflet and that lower membrane PS levels result in lower VLP production. Our computational findings indicate that PS may also have a direct influence on VP40 VLP assembly and budding, where a higher PS level will result in a higher VLP budding rate and filament dissociation rate. Our results further suggest that the assembly of VP40 filaments follow the nucleation-elongation theory, where initialization and oligomerization of VP40 are two distinct steps in the assembly process. Our findings advance the current understanding of VP40 VLP formation by identifying new possible mechanisms of PS influence on VP40 assembly. We propose that these mechanisms could inform treatment strategies targeting PS alone or in combination with other VP40 assembly steps.

Ebola virus (EBOV) was first identified in 1976 with two different outbreaks in Africa, where 284 and 318 people were infected with 53% and 88% mortality, respectively (1, 2). Since then, almost 30 known EBOV outbreaks have occurred, together causing more than 30,000 cases and 13,000 deaths (https://www.cdc.gov/vhf/ebola/history/distribution-map.html). Recent outbreaks in Uganda, Guinea, and the Democratic Republic of Congo illustrate the continued threat from this deadly infection.

Some therapies for EBOV disease (EVD) have shown promise in preclinical animal models (3, 4, 5, 6, 7, 8). Experimental treatments were also tested in humans during the multicountry outbreak in 2014 to 2016 including monoclonal antibody cocktails (9, 10), antiviral drugs (11, 12, 13, 14), and other therapies (15, 16, 17, 18, 19, 20, 21). Antibody cocktails and antiviral drugs have also been administered in the most recent EBOV epidemic in the Democratic Republic of Congo and, together with supportive care, reduced fatality rates (22, 23). However, most of these investigational treatments were given as Monitored Emergency Use of Unregistered and Investigational Interventions (MEURI) (22). Two monoclonal antibody therapies were recently approved for the treatment of EVD, but mortality remains high even with these treatments (more than 30%), and side-effects can be severe (24, 25, 26). Developing effective and safe EBOV therapies is challenging, in part, due to our limited understanding of key mechanisms of protein–protein and lipid–protein interactions in the EBOV life cycle.

Experimental EBOV studies are challenging because EBOV research is limited to facilities with biosafety level (BSL)-4 infrastructure. To enable EBOV life cycle studies in lower safety level laboratories, subsets of EBOV genes have been inserted into plasmids and expressed separately or together in transfected cells. Matrix protein VP40 is the main component of the EBOV matrix and is critical for EBOV assembly and budding. VP40, when expressed independently of the other six EBOV proteins, has been shown to form virus-like particles (VLPs) with similar size, shape, cell attachment, and entry properties as EBOV virions (27, 28, 29, 30). These noninfectious VP40 VLPs therefore represent a useful tool for studying EBOV assembly and budding processes *in vitro* in BSL-2 conditions.

This VP40 system has generated key insights into VP40 assembly. VP40 is known to form homodimers in the cytoplasm through N-terminal domain interactions, abrogation of which halts plasma membrane (PM) localization of VP40 and budding of VLPs (31). VP40 dimers bind to the cell membrane through interactions between VP40 C-terminal domains and phosphatidylserine (PS) (32). VP40 membrane dimers further assemble into hexamers and larger oligomers in the growing virus filaments (31, 33). There is evidence that the assembly and budding of VP40 VLPs is dependent on the level of PS in the host cell membrane (32, 34), suggesting that PS levels or VP40–PS interactions could be a drug target to disrupt EBOV reproduction.

Membrane PS levels have been shown to affect VP40 membrane binding (34), as well as impact the relative number of VP40 oligomers and VLPs produced over 48 h (34, 35). However, it remains unclear if the impact of PS on VP40 membrane association is able to account for the observed impacts on VP40 oligomer levels and VLP production or if additional PS-dependent mechanisms exist (Fig. 1). Furthermore, the dynamics of VP40 filament growth have not been reported, making it difficult to predict the long-term impacts of disrupting VLP assembly by targeting PS. These questions are critical for the development of EBOV treatment but difficult to answer using experimental approaches alone. It is not always possible to experimentally uncouple individual steps in the VLP assembly process (*e.g.*, VP40 membrane binding and oligomerization) or to obtain high temporal- and spatial-resolution data to quantify VP40 dynamics.

**Figure 1:**
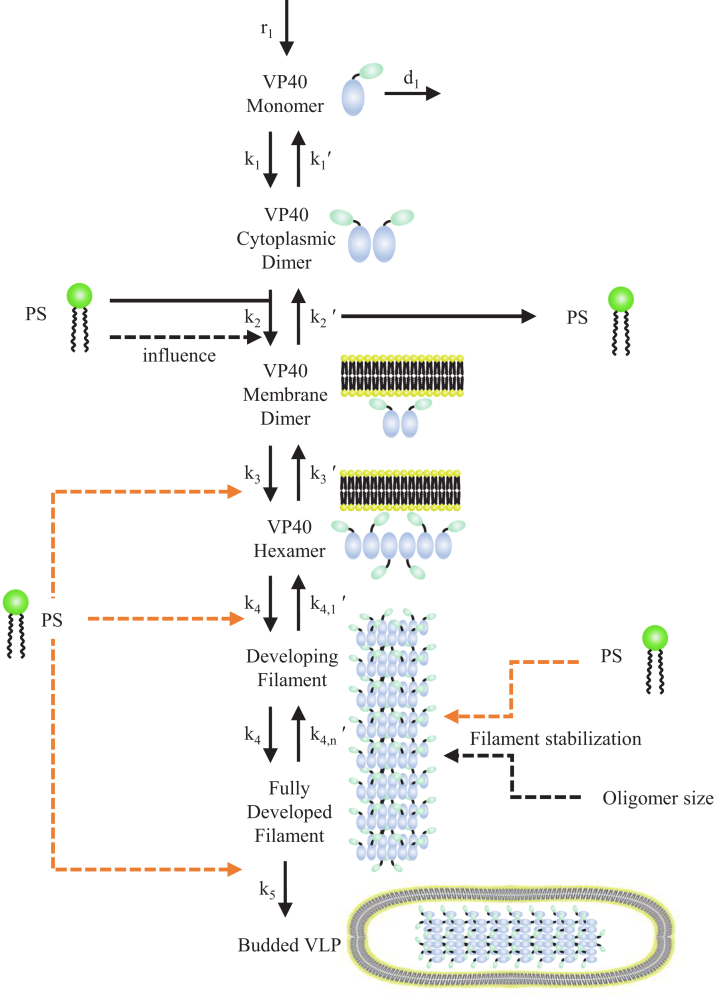
**Diagram of VP40–PS interactions and their incorporation into the ODE-model structure.** Our model incorporates VP40 dynamics spanning VP40 monomer production to VLP budding. PS is known to affect VP40 membrane association (k_2_, *black dashed line*), but it remains unclear if this is enough to explain experimentally observed differences in VLP filament growth profiles and VLP production under different PS concentrations (34, 35). In this work, we test if PS impacts on VP40 membrane association are sufficient to explain differences in filament growth and VLP production using our “preliminary” model. To explore other possible impacts of PS on VP40 VLP dynamics, we build progressively more complex models by including impacts of PS on filament stabilization (k_4,n_′), hexamer formation (k_3_), oligomerization (k_4_), and VLP budding (k_5_) (*orange dashed lines*) in our “stabilization,” “hexamer,” “filament,” and “budding” models, respectively. Details for each model are outlined in Table 1. ODE, ordinary differential equation; PS, phosphatidylserine; VLP, virus-like particle.

Computational models are complementary to experimental approaches. Computational models have been applied to evaluate viral infection dynamics in populations (36, 37), physiological disease progression and treatment efficacy (38, 39), viral replication, and cellular immune responses (40, 41). For EBOV, computational models have been applied on both population (42, 43, 44) and physiological (39, 45, 46, 47, 48) levels. In contrast, subcellular models for EBOV assembly are not currently available. However, emerging experimental data and a more detailed understanding of the EBOV replication cycle (34, 35) create an opportunity for a quantitative, computational characterization of EBOV assembly.

In this study, our goal is to identify potential mechanisms by which membrane PS levels affect VP40 VLP production. Toward this goal, we build computational models that represent a variety of biological hypotheses (Fig. 1). We then evaluate the ability of each of these computational models to reproduce experimental data. Through this evaluation against experimental data, the models will help identify the most likely biological hypotheses and guide future experimental studies.

We develop subcellular-level ordinary differential equation (ODE)–based models of EBOV VP40 assembly and budding. The models are built and calibrated using experimental data from VP40 studies (34, 49). We evaluate the performance of each computational model by comparing it to both qualitative and quantitative experimental observations. Model outputs and predictions include the dynamics of VP40 oligomer accumulation and filament growth, as well as the impact of PS on these processes.

Our work provides insights into the dynamics and robustness of VP40 oligomerization into VLPs, quantifies the influence of individual assembly steps on VLP production, and identifies potential mechanisms for PS influence on VLP production. These insights inform development of VP40-targeted and PS-targeted therapies for EVD.

## Results

### A novel ODE-based model of EBOV VP40 VLP assembly and budding

We first aim to determine if the known impact of PS on VP40 membrane association is sufficient to produce the experimentally observed changes in VP40 oligomer levels and VLP production. We therefore develop our “preliminary” model (Table 1), which includes the known influence of PS level on the dissociation constant (K_D2_) of VP40 dimer with the host cell membrane (32, 34).

**Table 1:** Model construction

Included mechanism	Model name				
	Preliminary	Stabilization	Hexamer	Filament	Budding
PS influence on VP40 membrane association	√	√	√	√	√
Filament stabilization		√	√	√	√
PS influence on filament stabilization		√	√	√	√
PS influence on hexamer formation			√		
PS influence on filament growth				√	
PS influence on budding rate					√

This “preliminary” model spans VP40 monomer production to VLP budding. VP40 monomers are produced and dimerize. VP40 dimer binds to the host cell membrane at a PS-dependent rate. Membrane bound VP40 dimers further combine to form hexamers. Hexamers serve as the building blocks for longer VP40 oligomers that form filaments. Fully developed filaments bud to form VLPs (Fig. 1).

The experimental data that we use to evaluate our computational models are described in detail in Experimental procedures, but the key observations are summarized here:

(1) Lower PS levels are associated with lower oligomer ratio.
(2) Transfected cells produce approximately 1 × 10^5^ VLPs per cell 24 h after transfection.
(3) The relative frequency of oligomers decreases from hexamers to 42-mers, and this decrease is more pronounced at lower PS levels.
(4) VLP production is reduced at lower PS levels, and this reduction is sustained over 48 h after transfection.

Our initial analysis aims to determine if the known impact of PS on VP40 membrane association is sufficient to produce the observed changes in VP40 oligomer levels and VLP production (34, 35). Our “preliminary” model (Table 1) therefore only includes the known influence of PS level on the dissociation constant (K_D2_) of VP40 dimer binding to cell membrane (32, 34). Our result indicates that the “preliminary” model is unable to reproduce the experimentally measured VLP production and oligomer frequencies simultaneously. The experimentally observed decrease in oligomer frequencies from hexamer to 42-mer can only be captured in the model when most VP40 have not bound to membrane, leading to no detectable VLP production (Fig. S1*A*). Conversely, when VLP production can be observed, the frequencies from hexamer to 42-mer become very similar (Fig. S1*B*).

These findings indicate that (a) our “preliminary” model is missing a key mechanism that would enable decreasing oligomer frequencies with oligomer size while producing VLPs and (b) PS impact on VP40 dimer membrane association alone is not sufficient to explain observed PS impacts on VLP production and oligomer frequencies.

### Filament stabilization is necessary to reproduce experimentally measured oligomer frequencies while producing VLPs

To address the inability of the “preliminary” model to match experimental results for VLP production and oligomer frequencies simultaneously, we introduce a filament stabilization mechanism.

We hypothesize that the physical structure and stability of a growing filament would be very different from an oligomer consisting of only a few hexamers. We therefore propose that VP40 hexamer association with existing oligomers would become stronger as the growing VP40 filament becomes larger. Mathematically, we represent this stabilization of the growing filament by decreasing the reverse rate constant of oligomerization (k_4,n_', Fig. 1) as the oligomer grows.

Addition of this stabilization mechanism enables the model to reproduce both decreasing oligomer frequencies and VLP production (Fig. S2, *A* and *B*). However, without direct influence of PS on the stabilization step, the model is unable to reproduce the experimental differences in oligomer frequencies among PS groups (Fig. S2*B*). The comparison of this model against the experimental data therefore indicates that PS influence on membrane association is insufficient to explain the observed impact of PS on oligomer frequencies. Thus, our results suggest that PS level could have a direct influence on filament stabilization.

**Figure 2:**
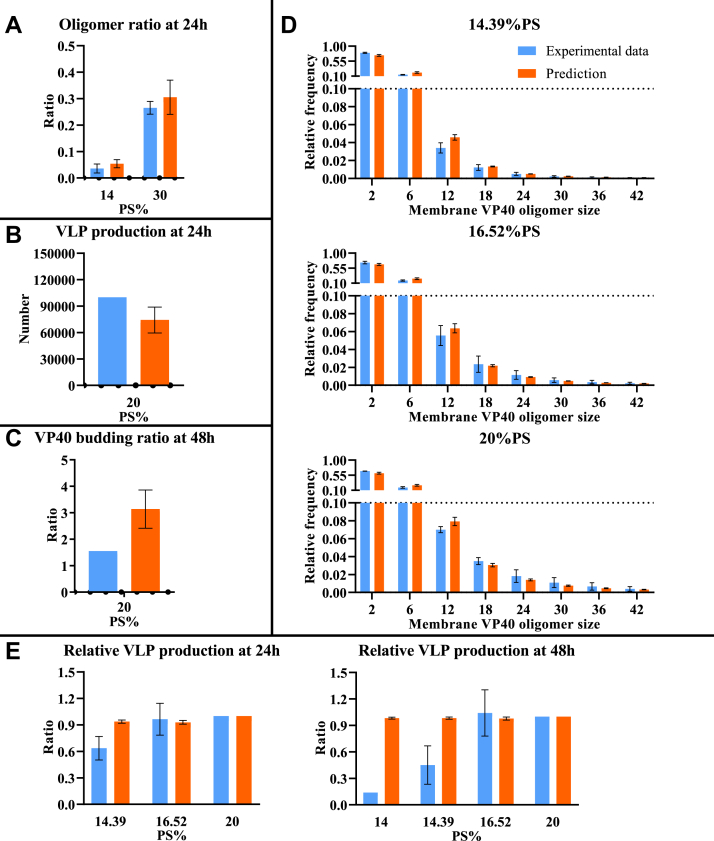
**Comparison between computational results from the “stabilization” model and experimental data.** Model fit is evaluated based on differences between simulated and experimentally measured values: *A*, oligomer ratio at 24 h. *B*, VLP production at 24 h. *C*, VP40 budding ratio at 48 h. *D*, oligomer frequency at 24 h for three different PS levels (14.39%, 16.52%, and 20%). *E*, relative VLP production at 24 and 48 h. Error bar indicates the SEM. Simulation data represent top five fits. Sample sizes of each experimental data are shown in Table S13. PS, phosphatidylserine; VLP, virus-like particle.

To test this possibility, we construct the “stabilization” model (Table 1) that includes filament stabilization as well as direct PS influence on this stabilization, where higher PS level leads to higher dissociation rate of hexamer from the growing filament. The “stabilization” model successfully reproduces the difference in oligomer ratio between the low and high PS groups (Fig. 2*A*), relative oligomer frequency among PS groups (Fig. 2*D*), and VLP production (Fig. 2*B*). The model slightly overestimates VP40 budding ratio compared to experimental data (Fig. 2*C*). Since this model can reproduce most of the experimental observations, this indicates that both stabilization and the influence of PS on this stabilization process are required.

**Figure 3:**
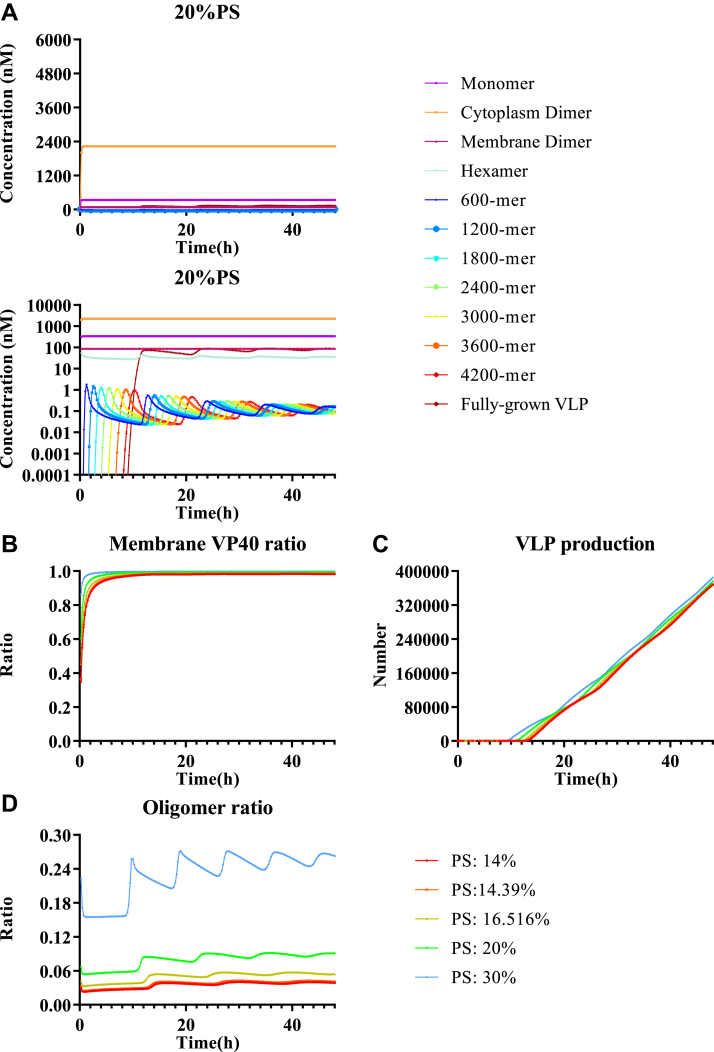
**Model predicted system dynamics of best fit in the “stabilization” model.** Using the calibrated “stabilization” model, we predict the following: *A*, time course of VP40 monomer and oligomers. The *upper subpanel* is on linear scale and the *lower subpanel* is on log scale. *B*, time course of membrane/cytoplasmic VP40 ratio. *C*, time course of VLP production. *D*, time course of oligomer ratio. VLP, virus-like particle.

Considering VLP assembly dynamics, our “stabilization” model predicts that the concentrations of monomer and dimer were increasing toward a steady state over time, while concentrations of hexamer and higher oligomers show fluctuations (Figs. 3*A* and S3). The fluctuations are caused by the dependence of the reverse rate constant $\left({\text{k}}_{4,\text{i}}^{\text{′}}\right)$ on filament size. Since this reverse rate constant decreases as the filament size increases, the transition of VP40 into smaller oligomers becomes out of balance with the transition of VP40 into the larger oligomers. Since larger oligomers are more stable than smaller oligomers in filament growth, VP40 temporarily preferentially accumulates either in oligomers that are close to the size of a full filament or the hexamer pool. Further, the decrease in reverse rate constant $\left({\text{k}}_{4,\text{i}}^{\text{′}}\right)$ with filament size means that equilibrium between smaller oligomers is established faster than equilibrium between larger oligomers. The variable size distribution of oligomers, plus the difference in equilibration time, together cause the wave-like patterns in filament growth.

Our model further indicates that dimer is the predominant form of VP40 in cytoplasm (Figs. 3*A* and S3), which is aligned with experimental observations (50). Although the concentration of cytoplasmic VP40 (monomer and dimer) is much higher than that of VP40 oligomers on the cell membrane, the overall amount of VP40 bound to the membrane is higher than the amount of VP40 in the cytoplasm due to the size and number of the oligomers (Fig. 3*B*). This is also aligned with experimental observations (34). These observations provide qualitative validation of our model predictions.

The “stabilization” model reproduces most of the experimental observations and captures the impact of PS on the oligomer ratio (Fig. 3*D*). However, the impact of PS on VLP production cannot be reproduced with this model (Fig. 3*C*). For the top five fits, model predictions show either identical VLP production among PS groups or differences due to fluctuations in time, and the impact of these fluctuations decreases with time (Fig. S4). As a result, the relative VLP production data is not well predicted from this model (Fig. 2*E*). The reason is that while VP40 monomer and cytoplasmic dimer concentrations change quickly with PS level, the number of membrane VP40 oligomers does not dramatically change (Fig. S3). Thus, these results indicate that our “stabilization” model is still missing key PS-related mechanisms. To systematically explore other potential PS-dependent mechanisms, we build three additional models: “hexamer,” “filament,” and “budding” models (Table 1). In the subsequent section, we describe addition of potential PS-dependent mechanisms in these models and evaluate the ability of these mechanisms to reproduce experimental data.

### Direct PS effect on VLP budding rate is required to reproduce longer term differences in experimentally measured VLP production

We build three additional extended models that include the influence of PS on VP40 hexamer formation (k_3_, “hexamer” model), filament growth (k_4_, “filament” model), and VLP budding (k_5_, “budding” model). We calibrate each of these models independently to experimental data. The ability of each model to reproduce the experimentally measured relative VLP production at 24 and 48 h indicates the feasibility of the relevant PS-influenced mechanisms.

**Figure 4:**
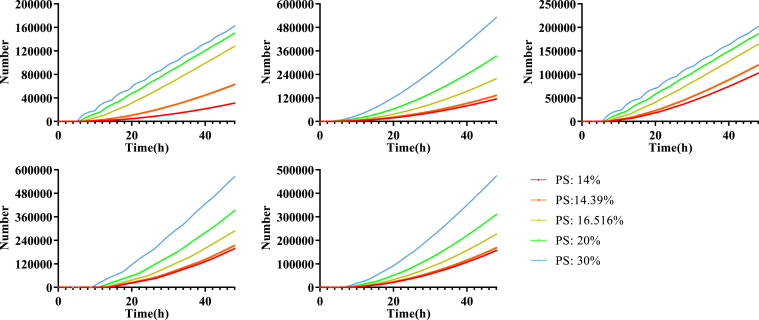
**Model predicted VLP production dynamics of the “budding” model.** Each panel represents one of the top five fits, and colors represent different PS levels. VLP productions for each PS concentration using the “budding” model are separated and different. PS, phosphatidylserine; VLP, virus-like particle.

**Figure 5:**
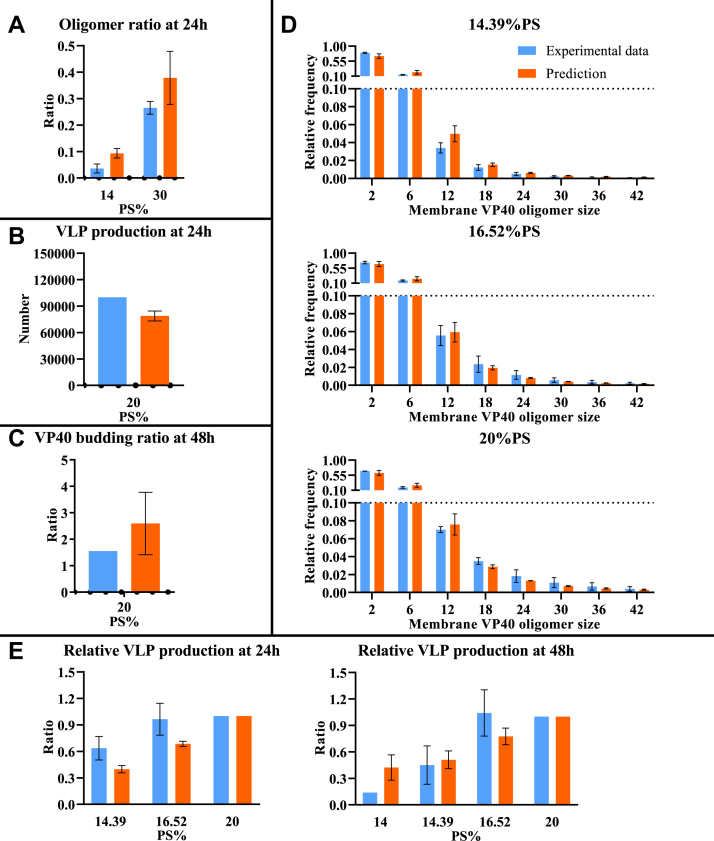
**Comparison between computational results from the “budding” model and experimental data.** Model fit is evaluated based on differences between simulated and experimentally measured: *A*, oligomer ratio at 24 h. *B*, VLP production at 24 h. *C*, VP40 budding ratio at 48 h. *D*, oligomer frequency at 24 h for three different PS levels (14.39%, 16.52%, and 20%). *E*, relative VLP production at 24 and 48 h. Error bar indicates the SEM. Simulation data represent top five fits. Sample sizes of each experimental data are shown in Table S13. PS, phosphatidylserine; VLP, virus-like particle.

When PS affects hexamer formation (k_3_, “hexamer” model) or filament growth (k_4_, “filament” model), the difference among PS levels remains small and mainly depends on fluctuations and the time point where VLPs starts budding (Figs. S5 and S6). No longer-term (48 h) effect on relative VLP production is observed. Thus the “hexamer” and “filament” model cannot fully reproduce the experimental data of relative VLP production, which indicates these mechanisms are still insufficient to represent the impact of PS on VLP production (Figs. S7 and S8). In contrast, when VLP budding (k_5_, “budding” model) is directly impacted by PS, consistent differences are observed over longer periods for each VLP budding curve (Fig. 4). This “budding” model was the only one that could match experimentally measured relative VLP production (Fig. 5*E*), while not losing accuracy in other predictions (Fig. 5, *A*–*D*).

**Figure 6:**
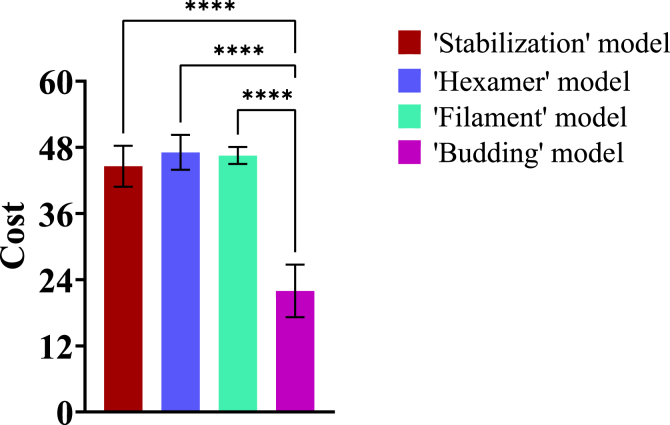
**Average cost of “stabilization,” “hexamer,” “filament,” and “budding” models.** Average costs for the top five fits are compared among models using ANOVA and there are statistically significant differences (*p*-value < 0.001) in cost among those models (Table S2). Least significant difference (LSD) is conducted to determine statistically significant pairwise differences, and statistical differences are identified between the “budding” model and other models (Table S3).

To quantify these observations and determine how well each model matches the experimental data, we determine the “cost” for each model. “Cost” represents the difference between model predictions and experimental data (a lower cost indicates that the model matches the experiments more closely). We define the cost as the sum of squared fold-change differences between experimental data and our simulation predictions (Equation 23). The average cost for the “budding” model is the lowest (22.0, Fig. 6, Tables S1 and S3) while the averages for the other three models are similar, indicating that the “budding” model matches the experimental data best.

All of our models assume that VP40 hexamers are the building units of VP40 assembly (27, 28, 34, 35, 49, 51, 52, 53). However, recent work indicates that the building block for VP40 VLPs can be dimers instead of hexamers (33). To assess the impact of this hypothesis, we modify our “budding” model to represent a dimer assembly system (Fig. S9). The predictions are similar to our hexamer assembly “budding” model (Fig. S10), indicating that our conclusions are independent of our assumptions about the specific building block of VP40 VLPs.

To validate our predictions before 24 h, we compare our simulations to experimentally measured VP40 membrane localization at both 8 h and 24 h. Though there are some differences between the absolute values of the simulations and experimental data (Fig. S11*A*), our simulations correctly predict that the membrane localization is nearly identical between 8 h and 24 h (Fig. S11*B*). Original data for membrane localization experiments are given in Tables S18 and S19.

Thus, our results show that the “budding” model is the only one that can reproduce all of our experimental data (Figs. 5 and S11). Taken together, our results indicate that (a) filament stabilization contributes to progressively decreasing oligomer frequencies in VP40 oligomers and the successful production of VLPs, (b) the difference in oligomer frequency is dependent on the membrane PS levels affecting filament stabilization, and (c) a direct influence of PS on mature filaments budding from the PM is important for the observed impacts of PS levels on VLP production.

### Sensitivity analysis identifies key mechanisms that can inform treatment development

With this “budding” model that can reproduce all of our experimental data, we next use global sensitivity analysis to determine which parameters are the main drivers of VLP production in the context of this complex system. To quantify the contribution of individual steps in VP40 assembly to VLP production and the influence of PS on the system, we use partial rank correlation coefficients (PRCCs) (54). PRCC measures the correlation between individual parameters and model outputs while correcting for the effects of variations in other parameters. The relationship between seven parameters (r_1_, k_1_, k_2_′, k_3_, k_4_, k_4,1_′, and k_5_) and four outputs (VLP production, oligomer ratio, relative VLP production, and VP40 budding ratio) are calculated.

The main output of interest is VLP production. Four key parameters (r_1_, k_4_, k_4,1_′, and k_5_) are identified to have significant positive PRCCs with VLP production at all PS levels (Table S6). Since r_1_ and k_5_ represent the “entry” and “exit” of VP40 in the system, respectively, their importance is expected. Filament growth parameters (k_4_ and k_4,1_′) that drive the process of VLP production are also significantly correlated. However, it is surprising that k_4,1_′, a reverse rate constant has a positive correlation coefficient with VLP. Local sensitivity analysis reveals that increasing k_4,1_′ leads to a significant increase in concentration of hexamer (Fig. S12). As a result, it creates a strong hexamer pool that can overcome the increase in the reverse rate constant and facilitate filament growth. This counterintuitively suggests that the decrease of reverse constant rate for filament growth could disrupt VLP production.

PRCC results for relative VLP production among different PS groups identify r_1_, k_2_′, k_4,1_′, and k_5_ as significant under low PS (14%) conditions at 24 h (Table S7). At 48 h, the parameters r_1_, k_2_′, k_4_, k_4,1_′, and k_5_ are significantly positively correlated with relative VLP production at low PS levels (Table S8). While r_1_, k_4_, k_4,1_′, and k_5_ are also positively correlated absolute VLP production, it suggests that targeting these parameters in combination with PS-targeted treatment may have synergistic effects in EBOV therapy.

Oligomer ratio is a metric of VP40 membrane binding and oligomerization. Since it represents the distribution of VP40 oligomers, any changes in the system might have an influence on it. As a result, almost all parameters are significantly correlated with the oligomer ratio (Table S9). PRCC results for VP40 budding ratio are similar to VLP production (Tables S6 and S10), as they both indicate the budding efficiency of VP40 VLPs.

Taken together, our sensitivity analysis indicates that the VLP assembly process is robust to disruptions in stability of filaments by allowing levels of VP40 hexamers to compensate for increases in the reverse rate constants for filament growth (k_4,1_′). Results also suggest that parameter influences can be different for different PS levels, possibly identifying opportunities for combination therapy development.

## Discussion

The dynamics of VP40 oligomer assembly into EBOV matrix remains unknown. While membrane PS level is known to affect VP40 membrane association and VLP production (34), the exact mechanisms of PS influence are still unclear. Using *in vitro* BSL-2 models (VP40 VLPs), integrated with computational modeling, we provide mechanistic insights into the role of PS in VP40 assembly into VLPs. We generate these insights by developing the first intracellular model describing EBOV VP40 assembly and budding dynamics.

Our simulations indicate that the rate constant for initialization of VP40 oligomerization is different from that of continuous oligomerization and that the growing filament structure stabilizes as it grows. While this stabilization effect has not been reported for VP40 filaments, our findings are consistent with the nucleation-elongation theory that has been established for other oligomers. For oligomerization of tubular or helical structures from single substrates, the initialization and elongation of the oligomerization are two different steps, and elongation is considered the faster step (55, 56). Nucleation-elongation mechanisms have been confirmed for microtubules (57, 58) and amyloid plaques (59, 60, 61, 62). Our results therefore suggest that this nucleation-elongation theory may also apply to VP40 oligomerization and can inform treatment strategies that target either nucleation or elongation steps.

Our prediction of preferential amplification of existing filaments (filament stabilization) is also consistent with structural studies showing VP40 to exist in a patchwork of assemblies at the PM inner leaflet (63, 64). These studies demonstrate that actin and VP40 diffused together and VP40 moved in a ballistic motion on these filaments in the absence of actin polymerization inhibitors (65). When actin polymerization is inhibited, VP40 exhibits constrained diffusion at the PM and VP40-enriched filaments emanating from the PM are significantly reduced (65). This transport mechanism of VP40 to sites of VLP assembly, in combination with our predicted nucleation-elongation kinetics of VP40 filament growth, would drive robust and effective VP40 assembly and budding.

One key finding of our study is that sustained differences in relative VLP production between PS groups can only be computationally reproduced when PS is directly affecting VLP budding. Furthermore, PS influence on filament stabilization is necessary for our models to reproduce relative oligomer frequency differences among PS groups. These two influences of PS (on VLP budding and filament stabilization) have not previously been reported. Our results indicate that PS might affect multiple steps in the viral budding process apart from the previously identified VP40 membrane association (34). Thus, disruption of PS–VP40 interactions could be a promising drug target.

Sensitivity analysis indicates that production of VLP is also highly dependent on VP40 production, VP40 membrane association, filament assembly, and VLP budding steps. Inhibiting those steps will decrease VLP production as well as relative VLP production at low PS level. Thus, while these steps could be good targets alone (66) (*e.g.*, using graphene (67)), a combination with PS targeting therapies such as fendiline (35, 68, 69, 70), staurosporine (71, 72), or bavituximab (73, 74) may have additional treatment efficacy.

Our model results show fluctuations in oligomer concentration, oligomer ratio, and mature VLP production for some parameter combinations. Biological evidence also exists for fluctuations in elongating structures, for example, in assembly of microtubules (75). Future experiments (*e.g.*, real-time imaging using total internal reflection microscopy as well as fluorescence correlation spectroscopy of VP40 at the PM interface and/or on supported lipid bilayers) can verify the existence of fluctuating dynamics in VP40 assembly. The phenomenon suggests the possibility of noise in experimental data from single time points, especially at single cell levels.

As with all computational models, some limitations apply to our model and analysis. We have not explicitly included transcription and translation processes, which may decrease our model-predicted time to steady state. The effects of diffusion or transportation are also not included in the model, and our rate constants thus represent effective rate constants. Our model currently does not include VP40 octamer rings, since it was not required for VLP production in previous studies (29, 31). Our model also successfully reproduces experimental data without VP40 octamers, which supports this prior conclusion. However, VP40 octamer is believed to play an unknown but important role in EBOV replication life cycle (29), which can be included in future models as more data emerge. Our model is based on and calibrated to the VP40 VLP system, and therefore, any impacts of the other EBOV proteins will need to be progressively incorporated as we and others work to translate our findings to live EBOV dynamics.

Another limitation is that experimental data are collected from different conditions, time points, and methods, leading to unavoidable differences between datasets. In some ways, this can be viewed as a strength as VP40-derived VLPs have been generated and used by many laboratories from different cell lines (HEK293, PSA-3, HUH7.5, A549, HeLa, CHO-K1, and others) (34, 76). Furthermore, the diversity in the data (Table S13) is exactly why computational models are a useful data integration tool. Overall, this study is focused on relative cost of model fits to determine the mechanisms needed to reproduce experimentally observed phenomena and trends.

In conclusion, we have built the first subcellular-level ODE-based model of the EBOV VP40 VLP system and integrated our model to data from *in vitro* VP40 VLP studies. Our computational approach enables complementary analyses that propose that PS may have direct influence on VP40 filament oligomerization, stabilization, and budding. Our combined experimental and computational approaches will enable further identification of key EBOV infection mechanisms and evaluation of treatment strategies.

## Experimental procedures

### ODE-based model construction

The structure of the hexamer-assembly based model is summarized in Figure 1. ODEs for the process are presented in Equations (1), (2), (3), (4), (5), (6), (7), (8). Total simulation time of the model is 48 h.

(1) $$\frac{\text{dA}}{\text{dt}}={\text{r}}_{1}-2{\text{k}}_{1}{\text{A}}^{2}+2{\text{k}}_{1}^{\text{′}}\text{B}-{\text{d}}_{1}\text{A}$$

(2) $$\frac{\text{dB}}{\text{dt}}={\text{k}}_{1}{\text{A}}^{2}-{\text{k}}_{1}^{\text{′}}\text{B}-{\text{k}}_{2}\text{BC′}+{\text{k}}_{2}^{\text{′}}\text{D}$$

(3) $$\frac{\text{dC}}{\text{dt}}={\text{r}}_{2}-{\text{d}}_{2}\text{C}-{\text{k}}_{2}\text{BC′}+{\text{k}}_{2}^{\text{′}}\text{D}$$

(4) $$\frac{\text{dD}}{\text{dt}}={\text{k}}_{2}\text{BC′}-{\text{k}}_{2}^{\text{′}}\text{D}-3{\text{k}}_{3}{\text{D}}^{3}+3{\text{k}}_{3}^{\text{′}}{\text{E}}_{1}$$

(5) $$\frac{{\text{dE}}_{1}}{\text{dt}}={\text{k}}_{3}{\text{D}}^{3}-{\text{k}}_{3}^{\text{′}}{\text{E}}_{1}-2{\text{k}}_{4}{\text{E}}_{1}^{2}-{\text{k}}_{4}{\text{E}}_{1}\sum_{\text{i}=2}^{\text{n}-1}{\text{E}}_{\text{i}}+2{\text{k}}_{4,1}^{\text{′}}{\text{E}}_{2}+\sum_{\text{i}=3}^{\text{n}}{\text{k}}_{4,\text{i}}^{\text{′}}{\text{E}}_{\text{i}}$$

(6) $$\frac{{\text{dE}}_{\text{i}}}{\text{dt}}={\text{k}}_{4}{\text{E}}_{1}{\text{E}}_{\text{i}-1}-{\text{k}}_{4,\text{i}-1}^{\text{′}}{\text{E}}_{\text{i}}-{\text{k}}_{4}{\text{E}}_{1}{\text{E}}_{\text{i}}+{\text{k}}_{4,\text{i}}^{\text{′}}{\text{E}}_{\text{i}+1}\left(1<\text{i}<\text{n}\right)$$

(7) $$\frac{{\text{dE}}_{\text{n}}}{\text{dt}}={\text{k}}_{4}{\text{E}}_{1}{\text{E}}_{\text{n}-1}-{\text{k}}_{4,\text{n}}^{\text{′}}{\text{E}}_{\text{n}}-{\text{k}}_{5}{\text{E}}_{\text{n}}$$

(8) $$\frac{\text{dF}}{\text{dt}}={\text{k}}_{5}{\text{E}}_{\text{n}}$$

Initial conditions:

A(0)=0

B(0)=0

$$\text{C}\left(0\right)=6.33\times 10^{7}\times \text{PS}\left(0\right)\text{/20}$$

D(0)=0

$${\text{E}}_{\text{i}}\left(0\right)=0\;\left(1\leq \text{i}\leq \text{n}\right)$$

F(0)=0

PS(0)=14%,14.39%,16.52%,20%,30% respectively

A: VP40 monomer in cytoplasm (nM).

B: VP40 dimer in cytoplasm (nM).

C: total PS (nM).

C′: PS available to interact with cytoplasmic VP40 dimer (nM, see Equation 12).

D: VP40 dimer on cell membrane (nM).

E(i): developing matrix protein consists of i VP40 hexamers (nM).

i: number of hexamers in developing filament.

n: number of hexamers in a mature filament. n = 770 in our model.

F: budded VLP (nM).

PS: total PS (%).

PS level will be updated by the concentration of C through Equation 9.

(9) $$\text{PS}\;=\;\frac{\text{C}}{6.33\;\times \;10^{7}}\;\times \;20$$

The dimer-assembly based model excluded the assembly of hexamer as a separate process (Fig. S9), and ODEs for the process are shown in Equations S1–S7 (See supporting information).

### Influence of PS on VP40 dimer binding to membrane

Data from a surface plasmon resonance (SPR) experiment on VP40–PS affinity are curve fitted to derive an equation for K_D_ as well as available PS as a function of PS concentration in the membrane during the membrane-binding process (35). K_D_ and PS available for the binding process are calculated through the following steps. We consider the following reversible binding reaction:

C′+B↔D

B: VP40 dimer in cytoplasm (nM).

C′: PS available to interact with cytoplasmic VP40 dimer (nM).

D: VP40 dimer on cell membrane (nM).

At steady state:

(10) $${\text{k}}_{2}\times \left(\text{C′}-\text{D}\right)\times \text{B}={\text{k}}_{2}^{\text{′}}\times \text{D}$$

(11) $$\text{D}=\frac{\text{C′}\times \text{B}}{\frac{{{\text{k}}_{2}}^{\prime }}{{\text{k}}_{2}}+\text{B}}=\frac{\text{C′}\times \text{B}}{{\text{K}}_{\text{D}2}+\text{B}}$$

K_D2_: equilibrium constant for VP40 dimer membrane binding (Table 2).

**Table 2:** Model parameters

Parameter	Value	Lower bound	Upper bound
k_1_	Calibrated	5 × 10^−4^/(nM s)	5 × 10^−2^/(nM s)
K_D1_	50^4^		
k_1_′	k_1_ × K_D1_		
k_2_	k_2_′/K_D2_		
K_D2_	Calculated according to PS, l, and m (Equation 13).		
k_2_′	Calibrated	2.5 × 10^−6^/s	2.5 × 10^−4^/s
k_3_	Calibrated^0^ Calibrated and calculated according to PS and x (Equation 22)^1^	7 × 10^−7^/(nM^2^ s)	7 × 10^−5^/(nM^2^ s)
K_D3_	200^4^		
k_3_′	k_3_ × K_D3_		
k_4_	Calibrated^0^ Calibrated and calculated according to PS and x (Equation 22)^2^	1.5 × 10^−4^/(nM s)	1.5 × 10^−2^/(nM s)
k_4,1_′	Calibrated	7 × 10^−2^/s	7/s
k_4,i_′	Calculated according to PS, i, y, t_1–3_, and u_1–3_ (Equation 20)		
k_5_	Calibrated^0^ Calibrated and calculated according to PS and x (Equation 22)^3^	7 × 10^−6^/s	7 × 10^−4^/s
r_1_	Calibrated	0.2 nM/s	20 nM/s
r_2_	d_2_ × C(0) (nM).		
d_1_	2.25 × 10^−5^ (78)		
d_2_	2.59 × 10^−5^ (79)		
n	770 (80, 81)		
x	Calibrated^1,2,3^	0	3.33
y	Calibrated	3	7
g	35.2, fitted		
h	1.48 × 10^4^, fitted		
l	9.20 × 10^−6^, fitted		
m	4.52 × 10^−4^, fitted		
t_1–3_	Fitted and calculated according to y		
u_1–3_	Fitted and calculated according to y		

0: ‘Stabilization’ model.

1, 2, 3: “Hexamer,” “Filament,” and “Budding” models.

4: Data from Dr Stahelin (available on request).

K_D2_ and C′ are fitted through Equation 11 using the SPR data (Table S11). Fitted values for K_D2_ and C′ at each PS level are included in Table S4.

Those SPR data are subsequently fitted into empirical Equations 12 and 13 to enable us to calculate C′ and K_D2_ values at different PS levels in our simulation.

(12) $$\text{C′}=\text{g}\times {\text{PS}}^{2}+\text{h}$$

(13) $${\text{K}}_{\text{D}2}=\frac{1}{\text{l}\times {\text{PS}}^{2}+\text{m}}$$

Fitted values of g, h, l, and m are included in Table 2.

### Influence of PS on filament stabilization

Filament stabilization is implemented by allowing the reverse rate constant to decrease as oligomerization increases until reaching a constant (nonzero) value. To account for PS influence on filament stabilization, the ratio of reverse rate constant of ith oligomer to hexamer is defined as f(i,PS) in Equation 14.

(14) $$\frac{{{\text{k}}_{4,\text{i}}}^{\prime }}{{{\text{k}}_{4,1}}^{\prime }}=f\left(\text{i},\text{PS}\right)=f_{\text{q}}\left(\text{PS}\right)\times {\text{exp}}^{f_{\text{s}}\left(\text{PS}\right)\times \text{i}}+f_{\text{o}}\left(\text{PS}\right)$$

i: number of hexamers in developing filament.

To include the influence of PS on this step, relative hexamer frequency data (transformed from the average of three replicates of oligomer frequency data) (35) is used to determine $f_{\text{o}}\left(\text{PS}\right)$, $f_{\text{q}}\left(\text{PS}\right)$, and $f_{\text{s}}\left(\text{PS}\right)$.

However, the frequency changing itself is not really the reverse rate constant for oligomerization. We only wish to have a trend of how k_4,i_′ decreased with oligomer size, not absolute values of how fast they decrease. Therefore, another parameter y is introduced to calculate a modified i in Equation 15.

(15) $$f_{i}\left(\text{y}\right)=\text{y}\times \left(\text{i}-1\right)+1$$

Combining Equations 13 with 14 we obtain Equation 15:

(16) $$f\left(\text{i},\text{PS}\right)=f_{\text{q}}\left(\text{PS}\right)\times {\text{exp}}^{f_{\text{s}}\left(\text{PS}\right)\times f_{i}\left(\text{y}\right)}+f_{\text{o}}\left(\text{PS}\right)$$

Values of $f_{\text{o}}\left(\text{PS}\right)$, $f_{\text{q}}\left(\text{PS}\right)$, and $f_{\text{s}}\left(\text{PS}\right)$ are then estimated by fitting Equation 16 to experimental relative oligomer frequency data at the PS levels that are measured (Table S12). To estimate $f_{\text{o}}\left(\text{PS}\right)$, $f_{\text{q}}\left(\text{PS}\right)$. and $f_{\text{s}}\left(\text{PS}\right)$ at all PS levels, we define their relationship with PS level by Equations (16), (17), (18), where t_1–3_ and u_1–3_ are fitted using measured PS levels and corresponding values of $f_{\text{o}}\left(\text{PS}\right)$, $f_{\text{q}}\left(\text{PS}\right)$, and $f_{\text{s}}\left(\text{PS}\right)$ (Table 2).

(17) $$f_{\text{o}}\left(\text{PS}\right)=\frac{{\text{t}}_{1}}{\text{PS}}+{\text{u}}_{1}$$

(18) $$f_{\text{q}}\left(\text{PS}\right)=\frac{{\text{t}}_{2}}{\text{PS}}+{\text{u}}_{2}$$

(19) $$f_{\text{s}}\left(\text{PS}\right)=\frac{{\text{t}}_{3}}{\text{PS}}+{\text{u}}_{3}$$

Combining Equations 14 and (17), (18), (19), we get Equation 20.

(20) $${{\text{k}}_{4,\text{i}}}^{\text{′}}=\left(\frac{{\text{t}}_{2}}{\text{PS}}+{\text{u}}_{2}\right)\times {\text{exp}}^{\left(\frac{{\text{t}}_{3}}{\text{PS}}+{\text{u}}_{3}\right)\times \text{y}\times \left(\left(\text{i}-1\right)+1\right)}+\left(\frac{{\text{t}}_{1}}{\text{PS}}+{\text{u}}_{1}\right)\times {{\text{k}}_{4,1}}^{\text{′}}$$

**Figure 7:**
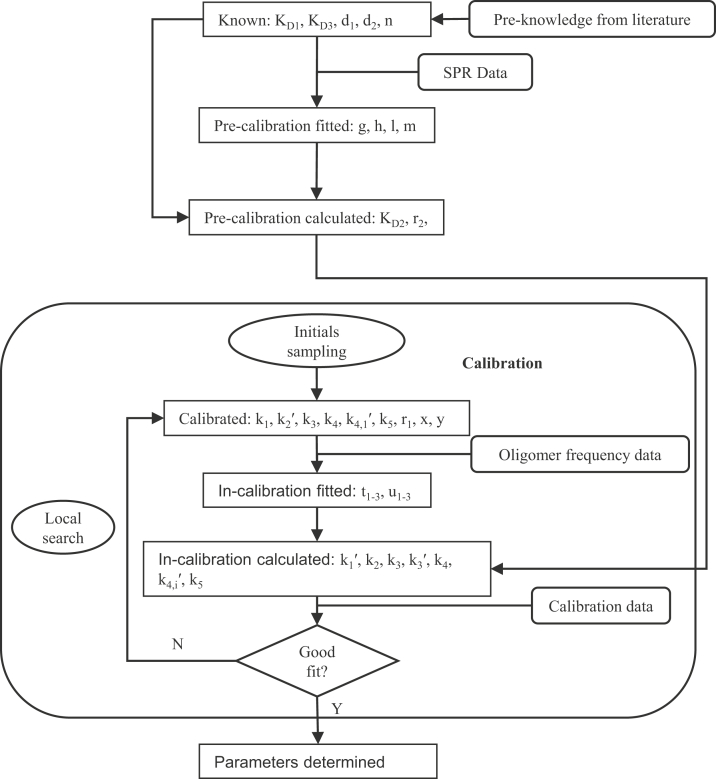
**Parameterization process.** All parameters can be organized into six categories, and most of them are determined in the calibration cycle. The in-calibration calculations of k_3_, k_4_, and k_5_ are only conducted in “hexamer,” “filament,” and “budding” models separately, and they will only undergo the first calibration process for the “stabilization” model.

The value of parameter y will be estimated during calibration (Fig. 7 and Table 2), and the estimation of t_1–3_ and u_1–3_ will be repeated for each calibration step. Finally we calculate k_4,i_′ by t_1–3_, u_1–3_, y, PS level, and i through Equation 20.

When stabilization is not influenced by PS level, Equation 20 is transformed into Equation 21

(21) $${{\text{k}}_{4,\text{i}}}^{\text{′}}=1.22\times {\text{exp}}^{-0.234\times \text{i}}+0.0298\times {{\text{k}}_{4,1}}^{\text{′}}$$

### Influence of PS on VP40 assembly and budding

Additional influences of PS on VP40 assembly and budding process are explored in three extended models using Equation 22

(22) $$\text{k}={\text{k}}_{\text{wt}}\times \left(1+\text{x}\times \left(\frac{\text{PS}}{20}-1\right)\right)$$

K_D2_: equilibrium constant for VP40 dimer membrane binding (Table 2).

k: involved parameter k_3_, k_4_, or k_5_ changing with PS level in “hexamer,” “filament,” or “budding” model separately.

k_wt_: involved parameter k_3_, k_4_, or k_5_ under 20% PS in “hexamer’, “filament,” or “budding” model separately.

Calibrated values can be found in Tables S14–S17.

### Experimental data used for model calibration and validation

We have five types of data under five different PS level available for model calibration (Table 3).

(1) VP40 oligomer ratio from PSA-3 and PSA-3 with PS supplementation groups. The oligomer ratio is defined as the ratio of VP40 hexamers or larger oligomers relative to dimers and monomers (34).
(2) VLP production number per cell from HEK293 cells at 24 h (35).
(3) Relative oligomer frequency from HEK293, HEK293 treated with 1 μM or 5 μM of fendiline (35), which was transformed from number and brightness (N&B) data and then averaged. Relative oligomer frequency is defined as the frequency of each VP40 oligomer, from membrane dimer to 42-mer, relative to the sum of oligomers (from membrane dimer to 42-mer). Concentration of larger oligomers was not experimentally detectable.
(4) Relative VLP production from HEK293, HEK293 treated with 1 μM or 5 μM of fendiline (35). Relative VLP production from PSA-3 group (34). Relative VLP production is defined as the amount of VLP in each group relative to WT PS levels.
(5) VP40 budding ratio from HEK293 cell line (49). VP40 budding ratio is defined as the ratio of VP40s in VLPs to VP40s in cell.

**Table 3:** Experimental data

PS level	14%	14.39%	16.52%	20%	30%
Experimental conditions	PSA-3 (genetic PS deficient)	HEK293 treated with 5 μM fendiline	HEK293 treated with 1 μM fendiline	HEK293 (WT)	PSA-3 supplied with additional PS
Oligomer ratio (24 h)	√				√
VLP production (24 h)				√	
Relative oligomer frequency (24 h)		√	√	√	
Relative VLP production (24 h)		√	√	√	
Relative VLP production (48 h)	√	√	√	√	
VP40 budding ratio (48 h)				√	

Details of data are included in Table S13.

Validation data include VP40 PM localization, which is defined as the percentage of membrane VP40 from dimer to 42-mer in the detectable VP40s including cytoplasmic VP40s. The data are generated from live cell-imaging experiments, performed at 8 h and 24 h post-transfection of WT enhanced green fluorescence protein-VP40 (EGFP-VP40) into HEK293 cells as previously described (35). HEK293 transfected cells were stained with Hoechst 3342 nuclear stain and a wheat germ agglutinin (Invitrogen Alexa Fluor 647) PM stain. Imaging was performed on a Nikon confocal microscope and image analysis was performed using Image J (imagej.nih.gov) to determine the percent PM localization for each time point. At least 24 cells were imaged per time point over three independent experiments on three different days.

### Parameter estimation and calibration

Model parameters are described in Table 2. The determination of parameters is outlined in Figure 7. Since there are limited data available on the value of rate constants from direct measurements, we calibrate the rate constants through available experimental data from literature and our own work. The model is implemented in MATLAB and solved using “ode15s.”

We calibrate our model using the available data (Table S13) to identify parameters that minimize the cost calculated by Equation 23.

(23) $$\text{cost}=\sum_{\text{j}=1}^{\text{N}}{\left(\left(\frac{\text{max}\left({\text{p}}_{\text{j}},{\text{e}}_{\text{j}}\right)}{\text{min}\left({\text{p}}_{\text{j}},{\text{e}}_{\text{j}}\right)}-1\right)\times {\text{w}}_{\text{j}}\right)}^{2}$$

N: total number of experimental data points

e_j_: jth experiment data

p_j_: jth model prediction

w_j_: weight assigned to jth data point (Table S5)

Calibration is done using MATLAB 2019b, where we use constrained nonlinear multivariable function solver (“fmincon”) for optimization and Latin hypercube sampling (“lhsdesign”) for sampling initial guesses (77). The sampling for r_1_, k_1_, k_2_′, k_3_, k_4_, k_4,1_′, and k_5_ are in log scale, while for x and y are linear scale. For each model, 50 initial guesses are generated. These initial guesses are used to initialize 50 independent optimizations and identify 50 parameter sets. Lower bounds and upper bounds used to sample initial guesses for each parameter are included in Table 2. The top five fits (out of 50) with lowest cost function values are analyzed (Tables S14–S17).

### Sensitivity analysis

PRCC is used to perform global sensitivity analysis (54) to quantify the impact of r_1_, k_1_, k_2_′, k_3_, k_4_, k_4,1_′, and k_5_ on various model outputs, including: VLP production, oligomer ratio, relative VLP production among PS groups, and VP40 budding ratio. PRCC ranks each parameter and target output and calculates the partial correlation coefficient between them while taking other parameter variations into account. PRCC is done with MATLAB 2019b, where we use partial correlation (“partialcorr”) for coefficient values calculation. Sampling range and method for r_1_, k_1_, k_2_′, k_3_, k_4_, k_4,1_′, and k_5_ is the same as calibration (Table 2). Empirical parameters “x” and “y” are fixed at 3.2 and 5, respectively, in order to focus our analysis on physiological parameters. Each parameter is sampled 500 times, and PRCCs are calculated for each model separately. *p* Values lower than 0.05 are regarded as significant.

Local sensitivity analysis is performed by fixing all parameters at values from the top fit of each model and varying the parameter of interest within two orders of magnitude.

## Data availability

The majority of the data are contained within the article and supporting information. For data not included within the article, data can be shared by contacting the corresponding author.

The code can be obtained from https://doi.org/10.5281/zenodo.5106604.

## Supporting information

This article contains supporting information.

## Conflict of interest

The authors declare that they have no conflicts of interest with the contents of this article.
