# Supplementary material for: Mechanisms of phosphatidylserine influence on viral production: A computational model of Ebola virus matrix protein assembly
Source: J Biol Chem. 2022 May 11;298(7):102025. doi: 10.1016/j.jbc.2022.102025 (PMC9218153; doi:10.1016/j.jbc.2022.102025)
Supplement: Supplemental Figures S1–S12 and Tables S1–S5 [file mmc5.docx]

**Supporting information: Mechanisms of phosphatidylserine influence on viral production: a computational model of Ebola virus matrix protein assembly**

Xiao Liu, Ethan J. Pappas, Monica L. Husby, Balindile B. Motsa, Robert V. Stahelin, Elsje Pienaar

**ODE-based dimer assembly model construction:**

Equation (S1)-(S7) represent the VP40 assembly pathway in the dimer model which assumes VP40 dimer to be the building block of filaments.

$\frac{\mathrm{dA}}{\mathrm{dt}}=r_{1}-2k_{1}A^{2}+2k_{1}^{'}B-d_{1}A$ (S1)

$\frac{\mathrm{dB}}{\mathrm{dt}}=k_{1}A^{2}-k_{1}^{'}B-k_{2}\mathrm{BC}'+k_{2}^{'}D_{1}$ (S2)

$\frac{\mathrm{dC}}{\mathrm{dt}}=r_{2}-d_{2}C-k_{2}\mathrm{BC}' +k_{2}^{'}D_{1}$ (S3)

$\frac{\mathrm{dD}_{1}}{\mathrm{dt}}=k_{2}BC'-k_{2}^{'}D_{1}-2k_{4}{D_{1}}^{2}-k_{4}D_{1}\sum_{i=2}^{n-1} D_{i}+2k_{4,1}^{'}D_{2}+\sum_{i=3}^{n} k_{4,i}^{'}D_{i}$ (S4)

$\frac{\mathrm{dD}_{i}}{\mathrm{dt}}=k_{4}D_{1}D_{i-1}-k_{4,i-1}^{'}D_{i}-k_{4}D_{1}D_{i}+k_{4,i}^{'}D_{i+1} (1<i<n)$ (S5)

$\frac{\mathrm{dD}_{n}}{\mathrm{dt}}=k_{4}D_{1}D_{n-1}-k_{4,n}^{'}D_{n}-k_{5}D_{n}$ (S6)

$\frac{\mathrm{dE}}{\mathrm{dt}}=k_{5}D_{n}$ (S7)

initial conditions:

$A\left( 0 \right)=0$

$B\left( 0 \right)=0$

$C\left( 0 \right)=6.33\times{10}^{7}\times\frac{\mathrm{PS}\left( 0 \right)}{20}$

$D_{i}\left( 0 \right)=0 (1\leq i\leq n)$

$E\left( 0 \right)=0$

$\mathrm{PS}\left( 0 \right)=14\%,14.39\%, 16.52\%,20\%,30\%$ respectively

A: VP40 monomer in cytoplasm (nM).

B: VP40 dimer in cytoplasm (nM).

C: Total phosphatidylserine (nM).

C′: Phosphatidylserine available to interact with cytoplasmic VP40 dimer (nM).

D_i_: Developing matrix protein consists of i VP40 dimers (nM).

i: Number of dimers in developing filament.

n: Number of dimers in a mature filament. n= 2310 in our model (nM).

E: Budded VLP (nM).

PS: Total phosphatidylserine (%).

**Figure S1. PS influence on VP40 membrane association is not sufficient to reproduce experimental measurements of both VLP production and relative oligomer frequency.** (A) When relative frequencies of lower oligomers (6-42mer) decrease, VLPs cannot be produced. (B) When VLPs are produced, the relative frequencies of lower oligomers are identical.


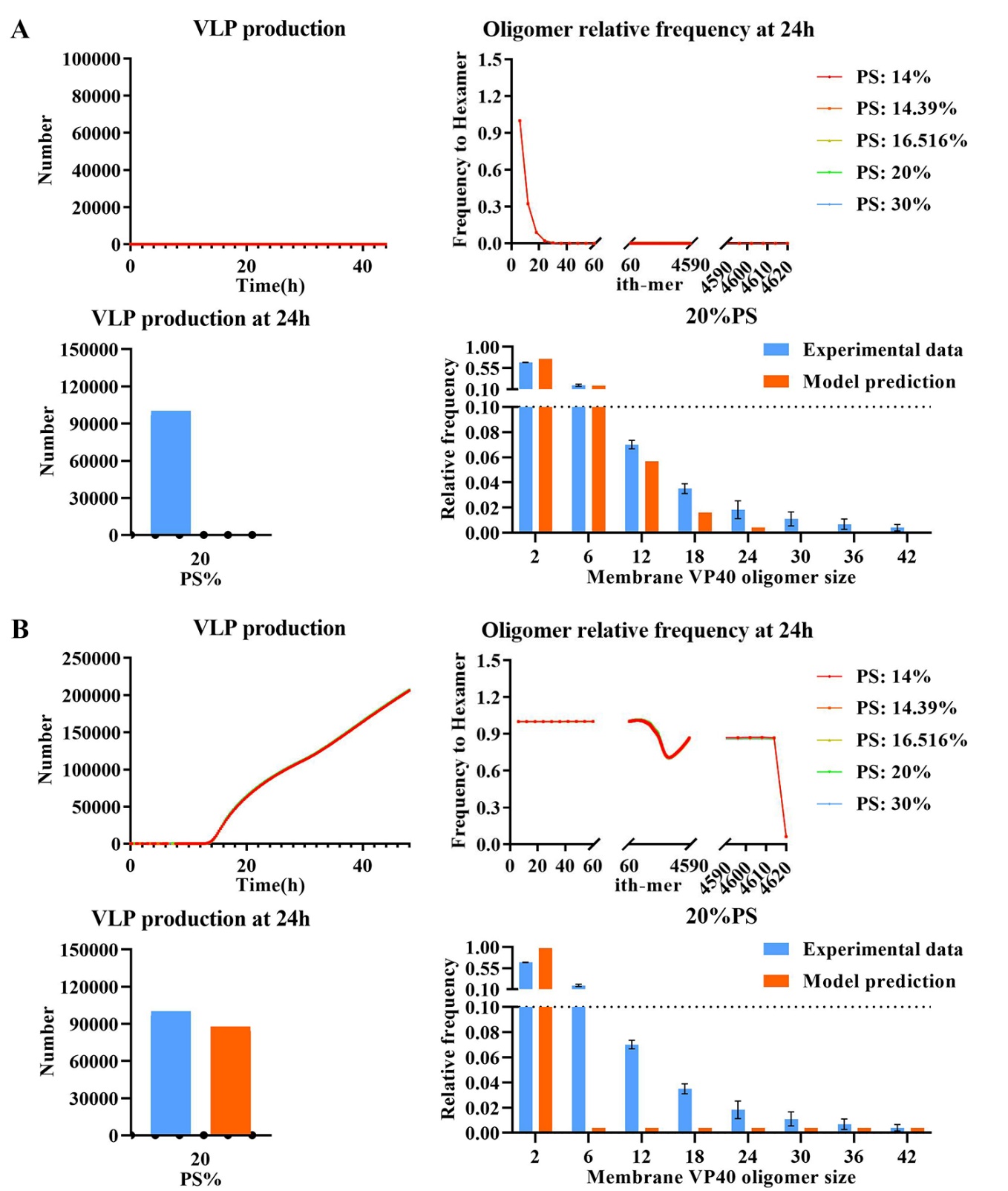


**Figure S2. VLP production and relative oligomer frequency prediction for model including filament stabilization.** Experimentally measured VLP production (A) and relative oligomer frequency (B) can be simultaneously reproduced by the model. Differences in relative oligomer frequency among PS groups cannot be reproduced.


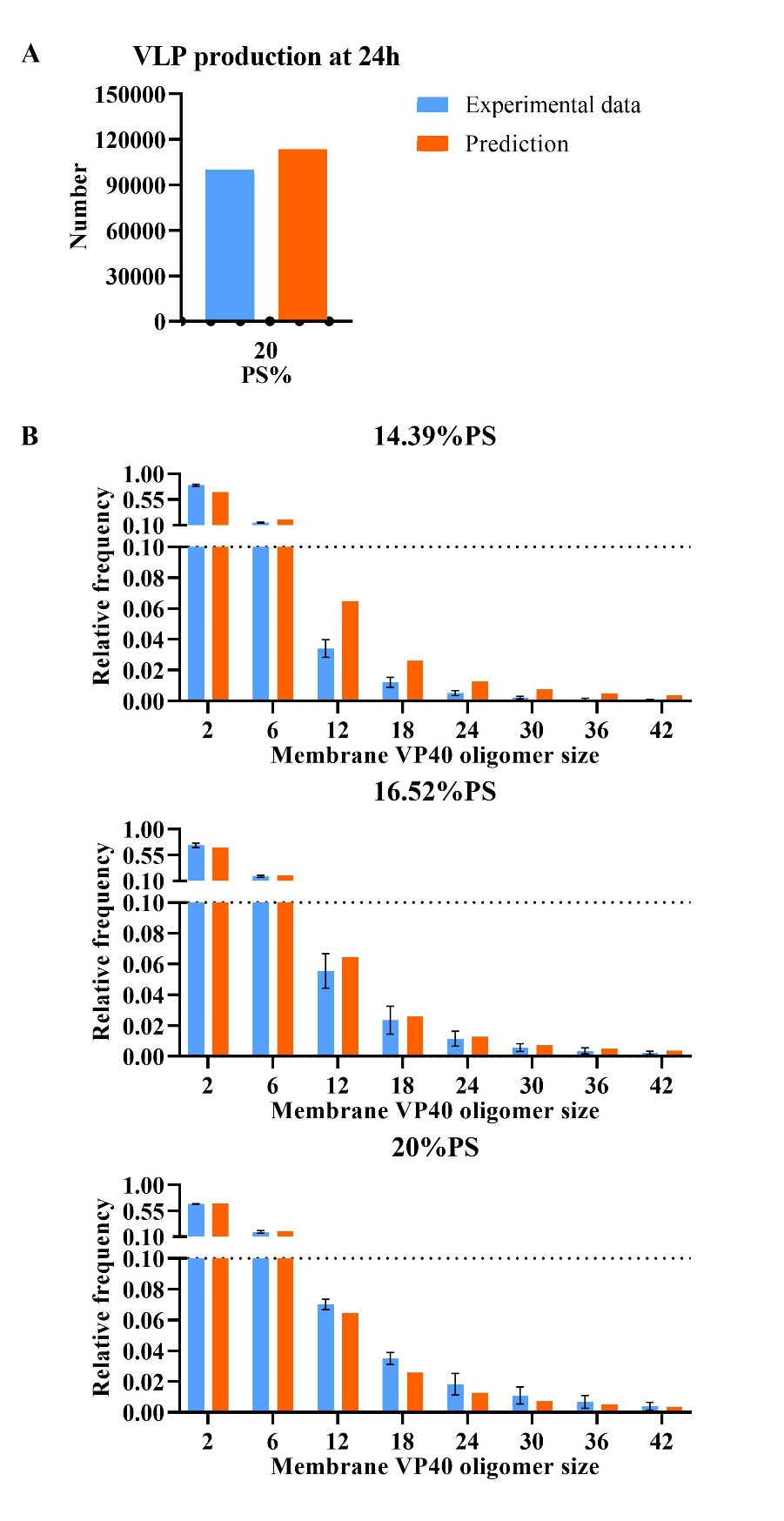


**Figure S3. Time course of VP40 monomer and oligomers of best fit in ‘Stabilization’ model.** PS level ranges from 14% to 30%. Left panels are on linear-scale and right panels are on log-scale. The panels under 20% PS are also used as an example to show the dynamics of VP40 in figure 3A.


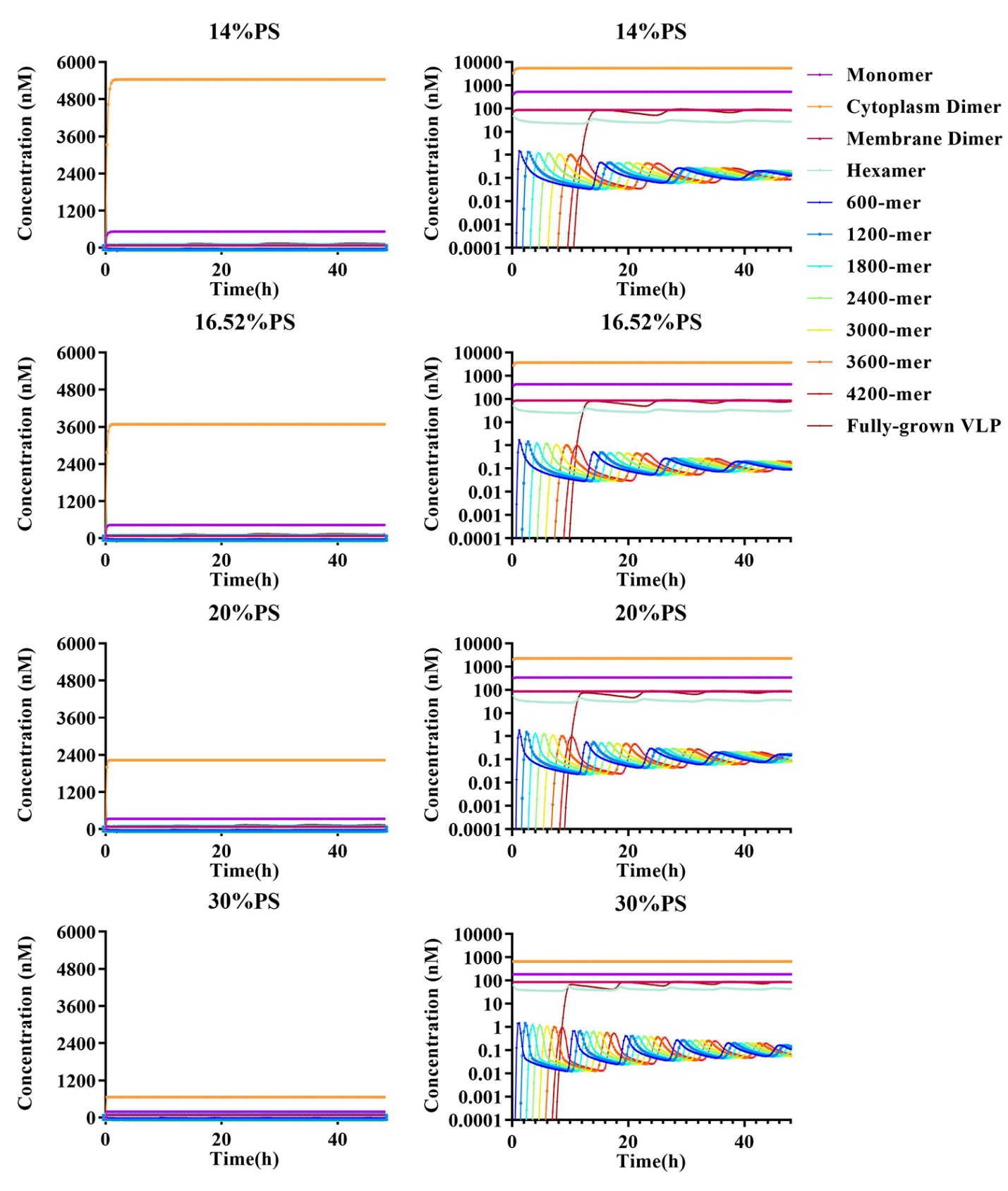

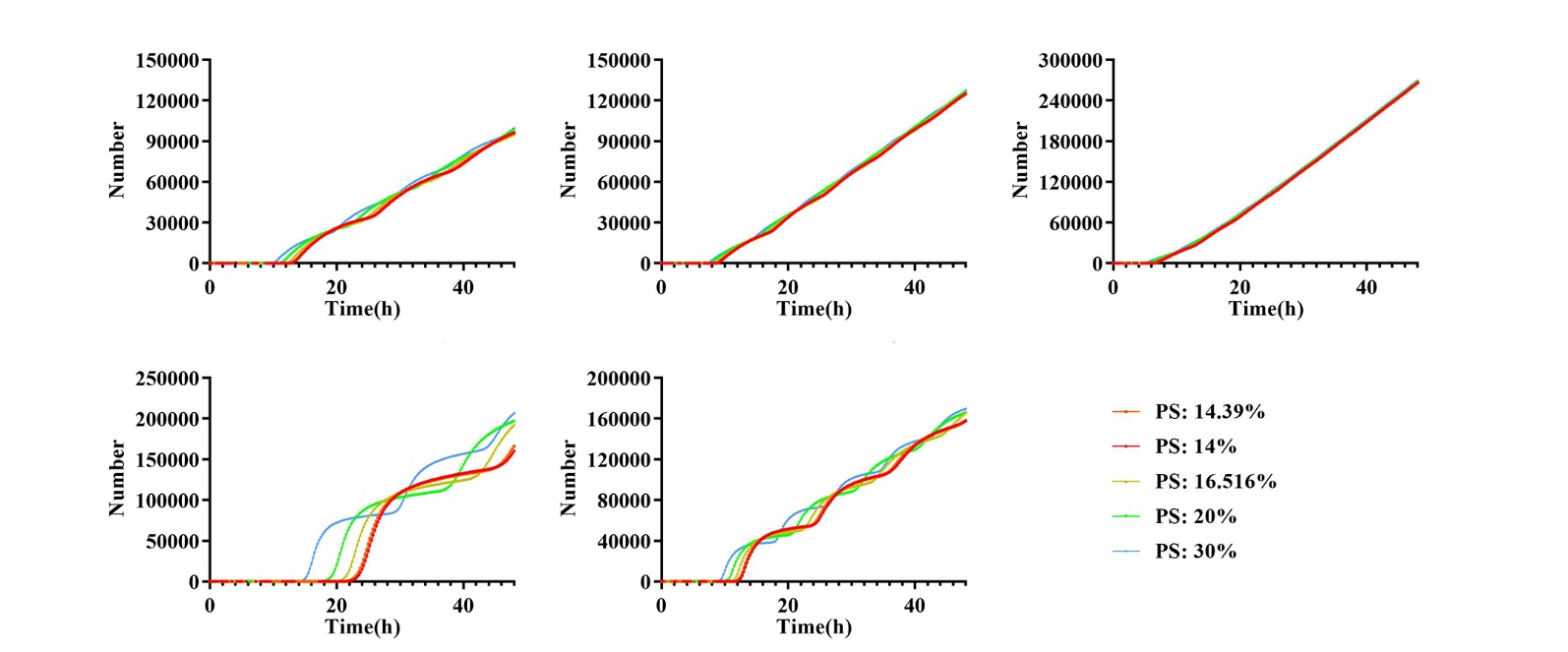


**Figure S5. VLP production dynamic of ‘Hexamer’ model from top 5 fits.** VLP production for each PS concentration under ‘Hexamer’ model are overlapping.

**Figure S4. VLP production dynamic of ‘Stabilization’ model from top5 fittings.** VLP production for each PS concentration under ‘Stabilization’ model are overlapping.


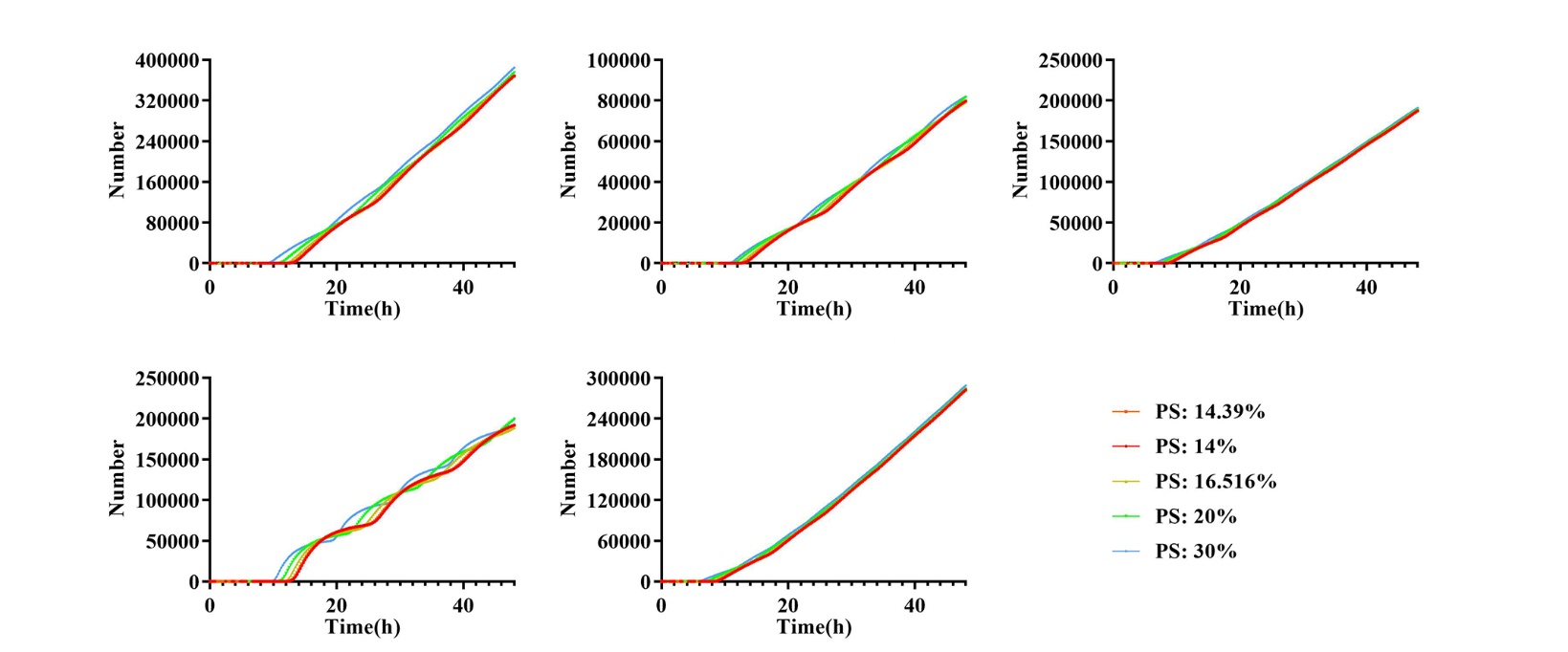


**Figure S6. VLP production dynamic of** **‘Filament’ model from top 5 fits.** VLP production for each PS concentration under ‘Filament’ model are overlapping.


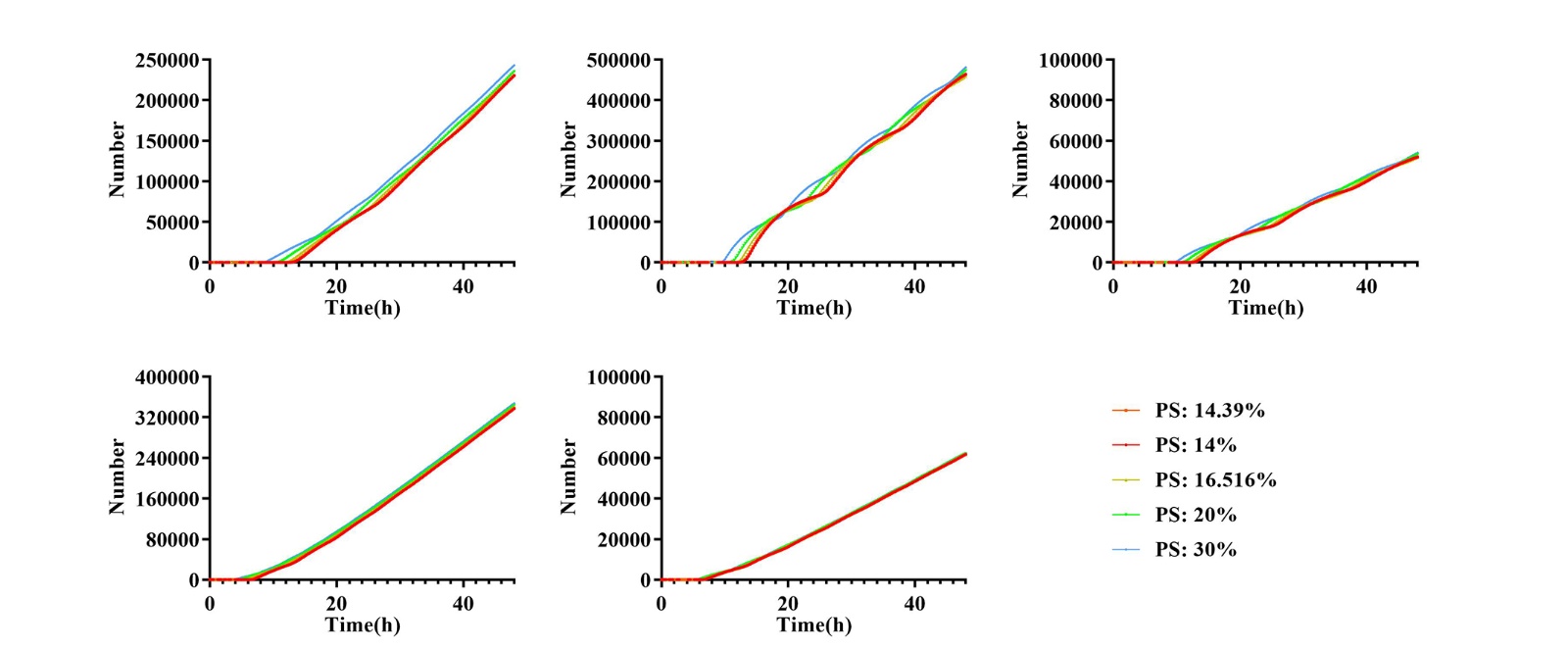

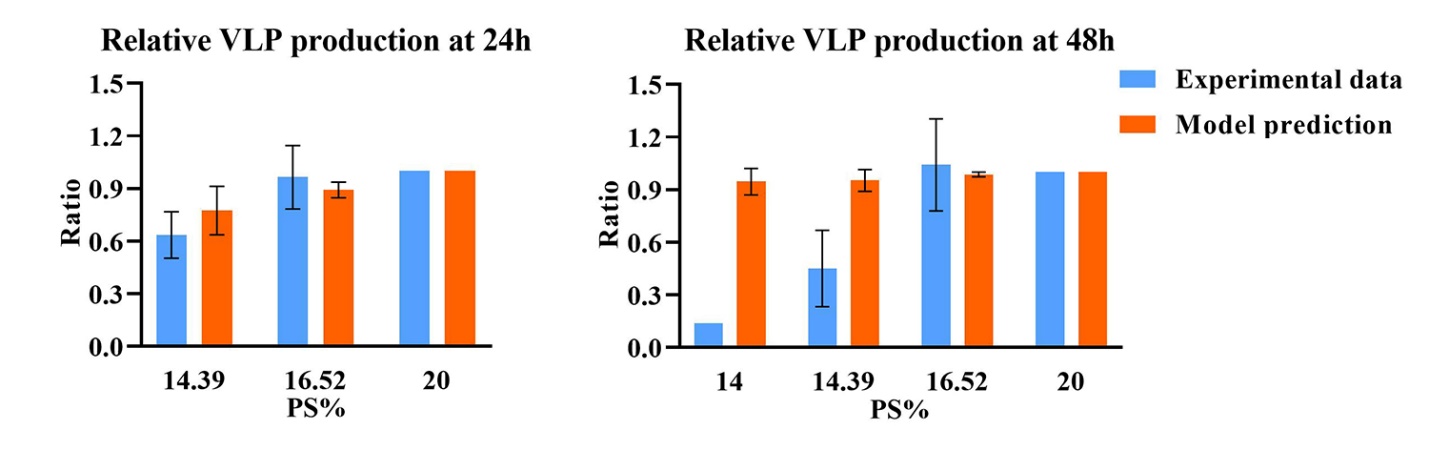


**Figure S7. Relative VLP production at 24 (left) and 48h (right) of ‘Hexamer’ model from top 5 fits.** No obvious difference can be observed in different PS groups, especially for 48h. Error bar indicates the SEM. Simulation data represents top 5 fits. Sample sizes of each experimental data are shown in Table S13.

**Figure S8. Relative VLP production 24 (left) and 48h (right) of ‘Filament’ model from top 5 fits.** No obvious difference can be observed in different PS groups, especially for 48h. Error bar indicates the SEM. Simulation data represents top 5 fits. Sample sizes of each experimental data are shown in Table S13.


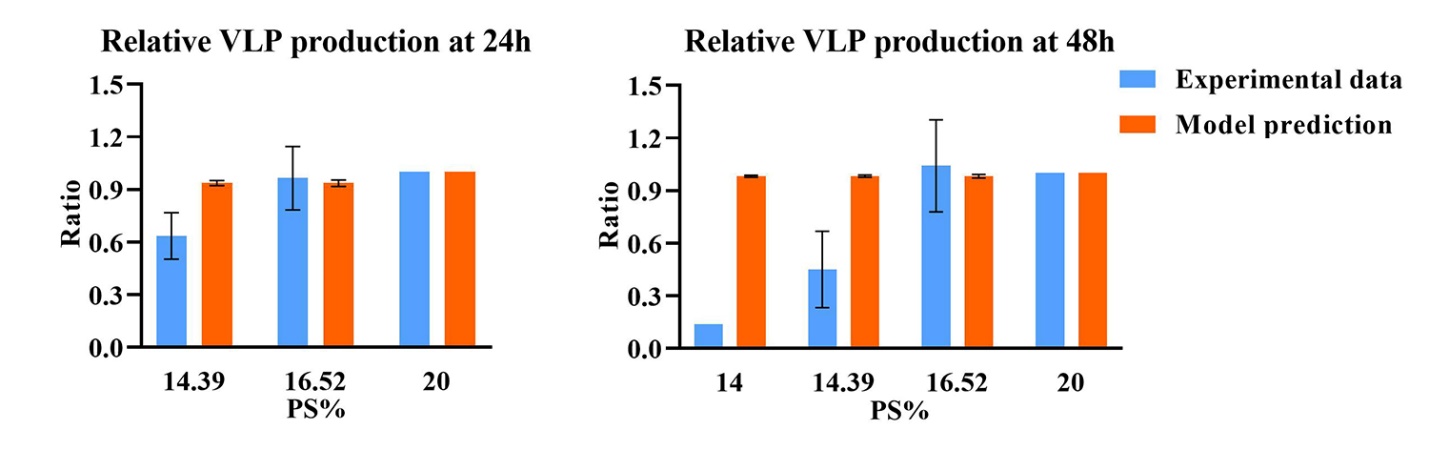


**Figure S9. Scheme of VP40 dimer assembly model.** The dimer assembly model is similar to hexamer assembly model. VP40 hexamer is deleted in this model, and filaments are directly built from VP40 membrane dimer. The model includes the influence of PS on VLP budding rate.


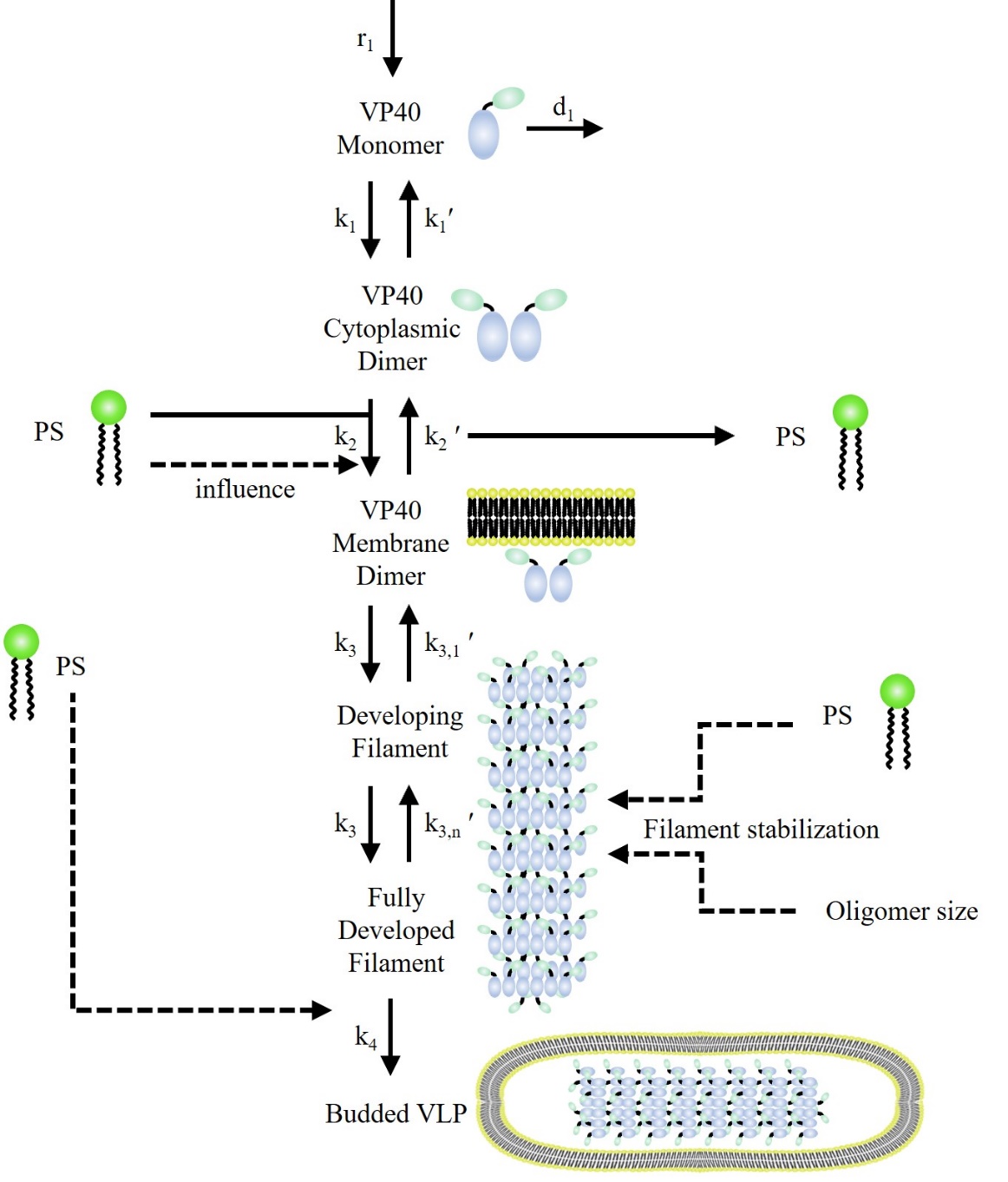


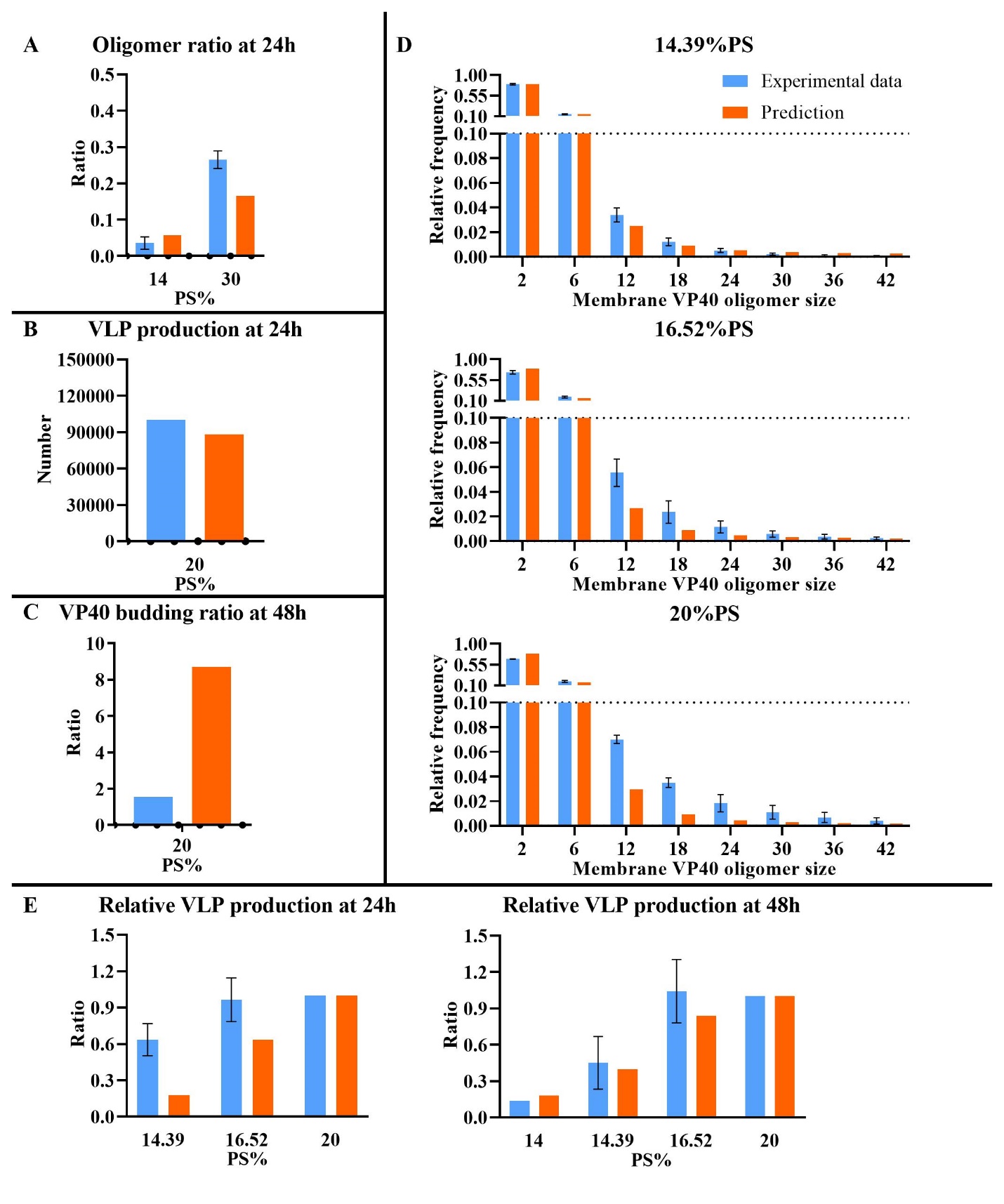


**Figure S10. Comparison between prediction and experiment data in dimer-based model (‘Budding’).** (A) Oligomer ratio. (B) VLP production. (C) VP40 budding ratio. (D) Oligomer frequency. (E) Relative VLP production. Error bar indicates the SEM. Sample sizes of each experimental data are shown in Table S13.


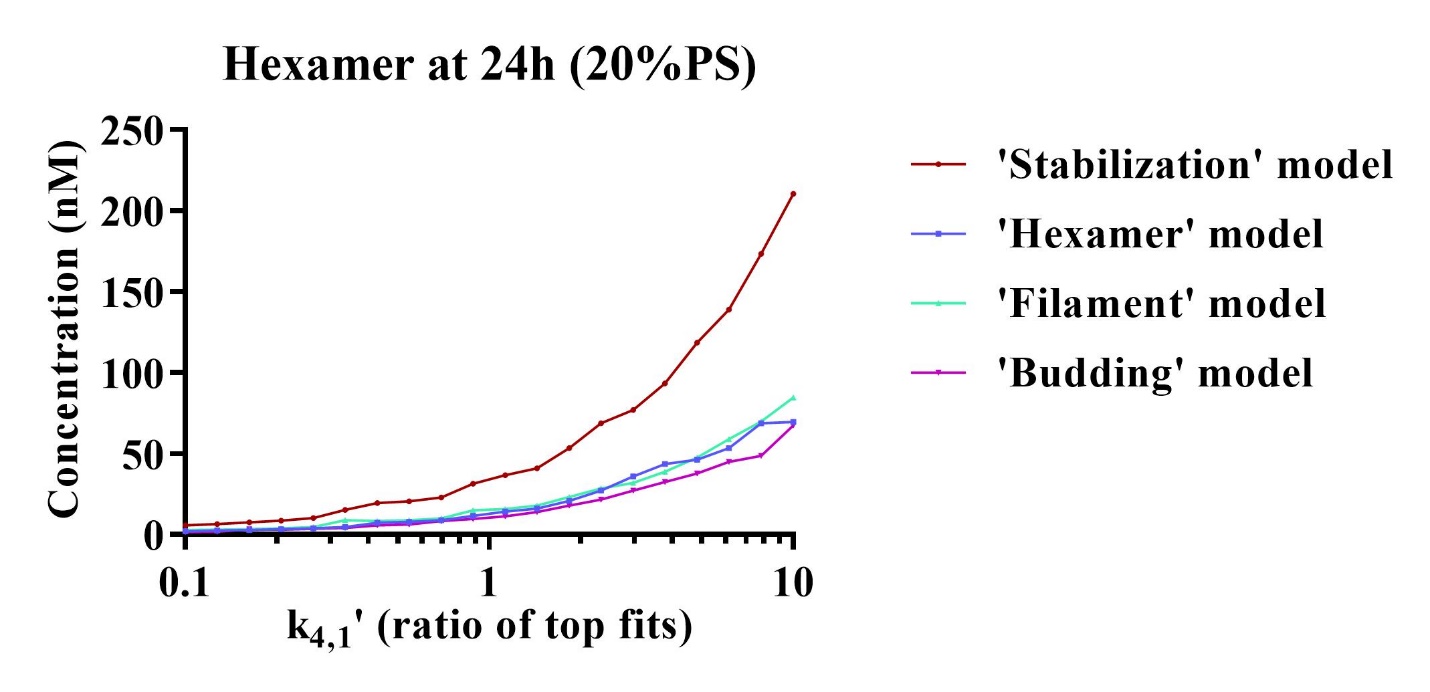


**Figure S12. Local sensitivity analysis of hexamer to k_4,1_′.** In each of the model, hexamer concentration is positively correlated to k_4,1_**′**.

**Figure S11. Verification of ‘Budding’ through VP40 membrane localization.** (A) Prediction of ‘Budding’ model and experimental data on VP40 membrane localization at both 8h and 24 h. (B) A ratio of VP40 membrane localization at 24h and 8h. Error bar indicates the SEM. Simulation data represents top 5 fits. Sample sizes of each experimental data are shown in Table S13.


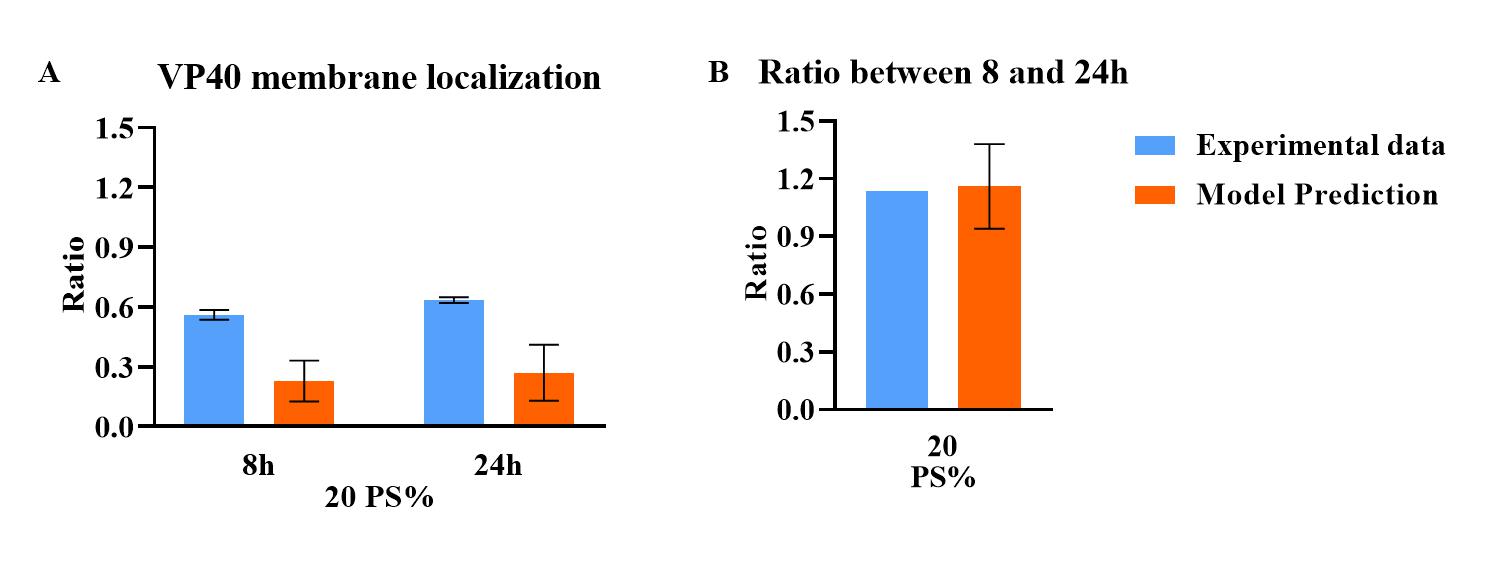


**Table S1. Top 5 lowest calibration cost for all models.**

| Top | ‘Stabilization’ | ‘Hexamer’ | ‘Filament’ | ‘Budding’ |
| --- | --- | --- | --- | --- |
| 1 | 40.2 | 44.0 | 43.8 | 15.7 |
| 2 | 41.9 | 45.0 | 47.0 | 20.0 |
| 3 | 44.8 | 45.9 | 47.0 | 22.4 |
| 4 | 46.4 | 48.7 | 47.2 | 23.0 |
| 5 | 49.6 | 51.8 | 47.7 | 28.7 |
| **Average** | **44.6** | **47.1** | **46.5** | **22.0** |

**Table S2. One-way ANOVA for cost among models.**

| ANOVA |  |  |  |  |  |  |
| --- | --- | --- | --- | --- | --- | --- |
| Source of Variation | SS | df | MS | F | **P-value** | F crit |
| Between Groups | 2.20×10^3^ | 3 | 7.32×10^2^ | 60.0 | **6.33×10^-9^** | 3.234 |
| Within Groups | 1.95×10^2^ | 16 | 12.2 |  |  |  |
| Total | 2.39×10^3^ | 19 |  |  |  |  |

**Table S3. LSD for cost between models.**

|  | t | p-value | significant |
| --- | --- | --- | --- |
| ‘Stabilization’ VS ‘Hexamer’ | -1.14 | 0.864 | N |
| ‘Stabilization’ VS ‘Filament’ | -0.881 | 0.804 | N |
| ‘Stabilization’ VS ‘Budding’ | 10.2 | 9.87×10^-9^ | Y |
| ‘Hexamer’ VS ‘Filament’ | -0.257 | 0.600 | N |
| ‘Hexamer’ VS ‘Budding’ | 11.1 | 3.07×10^-9^ | Y |
| ‘Filament’ VS ‘Budding’ | 11.4 | 2.22×10^-9^ | Y |

**Table S4. Fitted values of C and K_D2_**

| **PS (%)** | **C (nM)** | **K_D2_ (nM)** |
| --- | --- | --- |
| 1 | 1.76×10^4^ | 2.156×10^3^ |
| 11 | 1.54×10^4^ | 6.40×10^2^ |
| 22 | 3.27×10^4^ | 2.04×10^2^ |

**Table S5. Weight of calibration.**

| Data | w |
| --- | --- |
| VLP production number | 0.5^1^ |
| VP40 oligomer ratio | 0.8^2^ |
| Relative oligomer frequency | 1 |
| Relative VLP production | 1 |
| VP40 budding ratio | 1 |

1: Weight lowered because it is an estimated value.

2: Weight lowered because the interpretation of this data may not be accurate.

**Table S6. PRCC for VLP production.**

See “Supporting_information_PRCC.xlsx”

**Table S7. PRCC for relative VLP production at 24h.**

See “Supporting_information_PRCC.xlsx”

**Table S8. PRCC for relative VLP production at 48h.**

See “Supporting_information_PRCC.xlsx”

**Table S9. PRCC for Oligomer ratio.**

See “Supporting_information_PRCC.xlsx”

**Table S10. PRCC for VP40 budding ratio.**

See “Supporting_information_PRCC.xlsx”

**Table S11. SPR data.**

See “Supporting_information_data.xlsx”

**Table S12. Transformed relative oligomer frequency data for filament stabilization.**

See “Supporting_information_data.xlsx”

**Table S13. Data for calibration.**

See “Supporting_information_data.xlsx”

**Table S14. Calibration result for ‘Stabilization’ model.**

See “Supporting_information_calibration.xlsx”

**Table S15. Calibration result for ‘Hexamer’ model.**

See “Supporting_information_calibration.xlsx”

**Table S16. Calibration result for ‘Filament’ model.**

See “Supporting_information_calibration.xlsx”

**Table S17. Calibration result for ‘Budding’ model.**

See “Supporting_information_calibration.xlsx”

**Table S18. VP40 membrane localization at 8 h.**

See “Supporting_information_VP40_Membrane_localization.xlsx”

**Table S19. VP40 membrane localization at 24 h.**

See “Supporting_information_VP40_Membrane_localization.xlsx”
